# Identification of Specific Substances in the FAIMS Spectra of Complex Mixtures Using Deep Learning

**DOI:** 10.3390/s21186160

**Published:** 2021-09-14

**Authors:** Hua Li, Jiakai Pan, Hongda Zeng, Zhencheng Chen, Xiaoxia Du, Wenxiang Xiao

**Affiliations:** 1 Guangxi Colleges and Universities Key Laboratory of Biomedical Sensing and Intelligent Instrument, Guilin University of Electronic Technology, Guilin 541004, China; lihua@guet.edu.cn (H.L.); pan19970506@163.com (J.P.); zenghongda_518@163.com (H.Z.); chenzhcheng@163.com (Z.C.); 2 School of Life and Environmental Sciences, Guilin University of Electronic Technology, Guilin 541004, China

**Keywords:** FAIMS, deep learning, mixture, substance recognition, accuracy

## Abstract

High-field asymmetric ion mobility spectrometry (FAIMS) spectra of single chemicals are easy to interpret but identifying specific chemicals within complex mixtures is difficult. This paper demonstrates that the FAIMS system can detect specific chemicals in complex mixtures. A homemade FAIMS system is used to analyze pure ethanol, ethyl acetate, acetone, 4-methyl-2-pentanone, butanone, and their mixtures in order to create datasets. An EfficientNetV2 discriminant model was constructed, and a blind test set was used to verify whether the deep-learning model is capable of the required task. The results show that the pre-trained EfficientNetV2 model completed convergence at a learning rate of 0.1 as well as 200 iterations. Specific substances in complex mixtures can be effectively identified using the trained model and the homemade FAIMS system. Accuracies of 100%, 96.7%, and 86.7% are obtained for ethanol, ethyl acetate, and acetone in the blind test set, which are much higher than conventional methods. The deep learning network provides higher accuracy than traditional FAIMS spectral analysis methods. This simplifies the FAIMS spectral analysis process and contributes to further development of FAIMS systems.

## 1. Introduction

High-field asymmetric ion mobility spectrometry (FAIMS) is a new technology that uses nonlinear ion mobility variation under high electric fields to separate and recognize materials. FAIMS offers high sensitivity, fast detection speed, and miniaturization. Thus, it is expected to become an alternative to mass spectrometry (MS), ion mobility spectroscopy (IMS), and other analytical techniques [1,2,3,4].

FAIMS distinguishes ions using the differences between their ion mobility coefficients in low and high electric fields. When a sample is detected, FAIMS generates a unique chromatogram of the substance, which is called a fingerprint spectrum. Fingerprint spectra represent the compression of multiple FAIMS curves into a three-dimensional image, where the horizontal dimension represents the compensating voltage (CV), and the vertical dimension represents the radio frequency (RF) voltage. The intensity of the detected charged ions is presented using color.

Analysis of FAIMS spectra has become particularly important as FAIMS technology has developed. In the past, most FAIMS systems, including commercial equipment, focused on identification of individual chemical substances [5,6], such as benzene, toluene, and ethanol, for specific applications. Individual chemicals are distinguished well by their spectral shapes and numerical values. However, identifying specific chemicals within a mixture of substances is difficult because the ionic manifestations of different substances may overlap in a nonlinear manner and may be accompanied by the generation of new ions. In several cases, FAIMS will be used to analyze biological samples, including urine, feces, etc., which often contain a variety of volatile substances. Therefore, achieving substance-specific detection from complex mixtures is critical to further application of the FAIMS system.

Researchers have typically relied on a simple set of analysis tools that includes principal component analysis (PCA), linear discriminant analysis (LDA) [7,8,9,10], and extraction of image shapes. Such methods have achieved good results with regard to discriminant analysis of single substances [11,12] but cannot be implemented well in the analysis of mixtures. The research team of Cristina E. Davis used computer vision and natural language processing to address this problem and demonstrated the ability to maintain high levels of substance-specific identification despite the presence of other substances [13]. However, their method requires manual extraction of features from the data, which introduces a subjective element and is cumbersome.

Machine learning may provide an avenue for the analysis of FAIMS spectra. Recent discoveries in the fields of deep learning, computer vision, and natural language processing might be applied to analysis of FAIMS spectra. In particular, these advances may help to identify specific substances within mixtures. Deep learning has proven to be a great success in the field of computer vision. The proposed deep learning models such as AlexNet [14], GoogLeNet [15], VGG [16], ResNet [17], RNN [18], and LSTM [19,20] have enabled deep learning to show powerful performance in image classification, image detection, and sequence prediction. The deep learning models’ excellent performance has prompted researchers to apply them to a number of fields including medicine [21,22,23,24], agriculture [25,26,27], genomics [28,29,30], sentiment analysis [31,32,33,34], and knowledge graphs [35,36,37]. However, we have not yet seen the application of deep learning in the field of FAIMS. The purpose of this paper is to combine FAIMS and deep learning and to explore the possible application of deep learning to FAIMS spectral map analysis.

This study used the EfficientV2 model, which is cutting edge in the field of deep learning, to test spectra from a homemade FAIMS system. Abandoning traditional data processing methods such as wavelet transform and PCA dimensionality reduction, the collected spectral data were analyzed and judged in the presence of interfering chemicals. The final results showed that pre-trained deep learning network models can be established to identify specific chemicals in mixtures of substances. This supports the further application of FAIMS to practical detection, especially for analysis of biological samples that contain multiple substances [38].

## 2. Materials and Methods

### 2.1. FAIMS System

**Figure 1:**
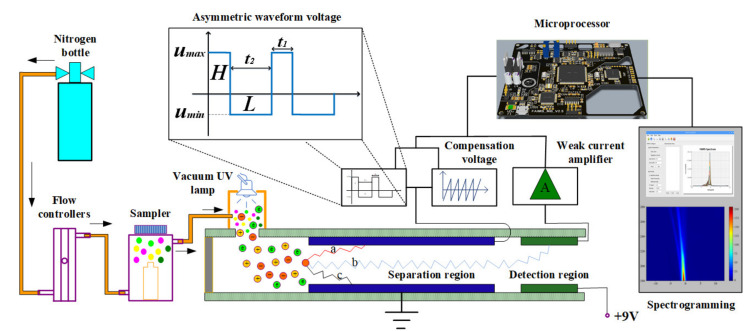
The homemade FAIMS experimental platform.

A homemade FAIMS system was used for sample acquisition. The entire FAIMS system is shown in Figure 1. The system was composed of a sampler module, FAIMS chip, weak current detector, power module, microprocessor controller, and spectrum display module. The carrier gas blew the sample through the sampler and into the ionization zone. After ionization by the 10.6 eV photo-discharge UV lamp (PKS106, Heraeus Co., Ltd., Hanau, Germany), the charged ions entered the migration zone.

The separation region of the homemade FAIMS experimental platform is composed of two parallel copper plates, with a size of 15 mm × 10 mm, which is the key structure to achieve ion separation. By applying high-field asymmetric waveform voltage and compensation voltage, the ions can pass through the separation region horizontally and then reach the detection region.

When the electric field intensity is less than 10,000 V/cm, the ion mobility basically does not change with the change of electric field intensity. However, when the ion is in a high-field condition (E > 10,000 V/cm), the ion mobility will change with the change of electric field intensity and presents a nonlinear change trend. 

The asymmetric square waveform applied on the separation region is shown in Figure 1 (L and H). The maximum voltage is represented by $U_{\max}$, and the minimum voltage is represented by $U_{\min}$ Since the distance between the separation region is constant (0.2 mm), the voltage can be represented by electric field strength. That is, the maximum value of electric field strength is $E_{\max}$ and the minimum value is $E_{\min}$. It is assumed that the movement time of ions under high-field conditions is $t_{1}$, the ion mobility is $k_{1}$, the movement time under low-field conditions is $t_{2}$, the ion mobility is $k_{2}$, and it satisfies the formula (1):

(1) $$E_{\max}t_{1}=E_{\min}t_{2}=H=L$$

Then the distance moved by ions in a period can be expressed as follows:

(2) $$\Delta S=S_{1}-S_{2}=v_{1}t_{1}-v_{2}t_{2}=k_{1}E_{\max}t_{1}-k_{2}E_{\min}t_{2}=(k_{1}-k_{2})H$$

In formula (2), $v_{1}$ and $S_{1}$ represent the velocity and displacement of ions in a high field, $v_{2}$ and $S_{2}$ represent the velocity and displacement of ions in a low field, and ion mobility k=v/E.

As the high-field asymmetric square waveform load has a relatively fast frequency (1 MHz), the ions in the separation region will have multiple tiny displacements superimposed, and eventually the ions will collide with the plate and be submerged, as shown in Figure 1 (ions a and c).

When a high-field asymmetric square waveform and a compensation voltage is applied to the separation region at the same time, the displacement difference of the ions within a period is neutralized, so that the ion can reach the detection region through the separation region horizontally, as shown in Figure 1 (ion b).

Different ions correspond to different ion mobility, resulting in different displacement differences. Finally, specific ions can be screened by modifying the displacement differences with specific compensation voltage values.

The ions that reached the detection zone were amplified by the weak current detector and transmitted to the microprocessor controller. The microprocessor controller communicated with the host computer via a serial port. The host computer software was written in Qt and communicated with the host computer via a serial port. When a sample was tested, the data acquisition command was sent to the host computer by clicking “Start.” The host computer received the CV and weak current data via the serial port and displayed a single curve that represented the sample in the spectral display area in real time. After one data acquisition process was completed, the host computer entered the idle state, changed the RF voltage, and repeated the above operation to complete plotting of multiple FAIMS curves. After all operations were completed, the data were added to the database and exported to the data table to facilitate data management. The plot function enabled the plotting of FAIMS spectra for subsequent data analysis and processing.

### 2.2. Sample Information

Ethanol (Xilong Chemical Co., Ltd., Shantou, China, 99.7%), ethyl acetate (Guangfu Technology Development Co., Ltd., Tianjin, China, 99.5%), acetone (Xilong Chemical Co., Ltd., Shantou, China, 99.5%), 2-butanone (Xilong Chemical Co., Ltd., Shantou, China, 99.5%), and 4-methyl-2-pentanone (Xilong Chemical Co., Ltd., Shantou, China, 99.0%) were used as experimental samples. A mixture was made by mixing equal volumes of the above five pure compounds. The pure compounds and their mixtures were reconfigured before each experiment.

### 2.3. Experimental Protocol

Pure compound data acquisition: 0.5 mL of a pure compound was placed in brown glassware using a pipette. The container was then put into a sampler using high-purity nitrogen (Ruida Chemical Technology Co., Ltd., Nanning, China, 99.999%) as the carrier gas. The carrier gas blew the volatile vapor from the headspace of the liquid sample into the FAIMS instrument for detection. In the homemade FAIMS system, the RF voltage was ramped from 180 V to 280 V in 10 V steps. The range of the compensating voltage was −13 V–+13 V and 1000 sampling points were used. Each sample took approximately 4–5 min to measure. Before each sample change, the sampler was purged with the carrier gas and the vacant sampler was collected to ensure that the FAIMS system was not contaminated with the original sample.

Mixed substance data acquisition: 0.5 mL of each pure compound was placed into brown glassware using a pipette, shaken and mixed well, and assayed as above. A total of 295 spectra were collected during the experiment. All spectra were collected within the same month and at a temperature of 25 °C to ensure the generalizability of the data. Fresh pure compounds and mixtures were used for each experiment.

The carrier gas flow rate through the sampler was controlled to 1500 mL/min using a D08-1F flowmeter (Sevenstar Electronic Technology Co., Ltd., Beijing, China). The carrier gas flow rate affects the acquired FAIMS spectra because higher flow rates blow larger quantities of volatile samples into the FAIMS system per unit of time. This leads to increases in the detected current. Therefore, all experimental conditions, except for the choice of samples, were designed to be consistent. The carrier gas flow rate was 1500 mL/min, the ballast resistance was 6 MΩ, and the RF voltage step was 10 V.

Computer software written in Qt was used to collect data and draw the spectra. The resolution of the spectra could be set. Higher resolutions could provide more effective information but lead to long model training times. The spectra resolution used in the experiments was 479 × 381. Python 3.7 was used as the programming language and Pytorch 1.7.0 [39] was the deep learning framework used with the model.

### 2.4. Principal Component Analysis and Support Vector Machine

Principal component analysis (PCA) is a commonly used method for data dimensionality reduction. It generates low-dimensional new variables by linear combination of high-dimensional original variables, and the new variables reflect the signal information of the original variables to the maximum extent [40,41,42]. Support vector machine (SVM) is an effective traditional classification model. It uses mathematical methods to find the best decision surface in a sample, thus separating the sample data [43,44,45].

### 2.5. Deep Learning Network

**Figure 2:**
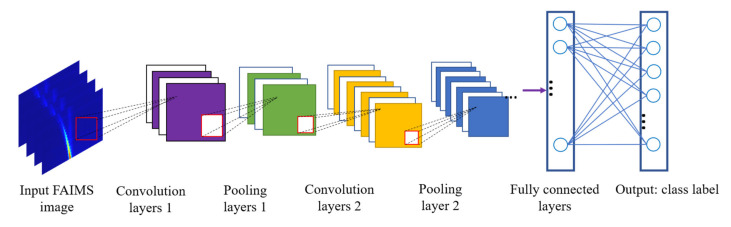
The structure of a typical convolutional neural network.

A convolutional neural network (CNN) is a type of deep-learning algorithm that has proven to be quite successful in the field of computer vision. Unlike traditional FAIMS spectral analysis methods (PCA or wavelet analysis), CNNs first rely on convolution layers to extract image features, followed perform down-sampling operations by pooling layers (this improves the efficiency and robustness of the model), and the data eventually reaches the output layer via the activation function. The output layer converts the probabilities for each class label by a logical function. The class label with the highest probability is the output result [46,47]. CNNs have no need to artificially select features that are subjectively important, which increases the objectivity of the results to some extent. Figure 2 represents the basic structure of a CNN.

In CNNs, the depth of the network, the width of the network, and the input image resolution largely determine the performance of the model. Researchers have been exploring the best architectures for CNNs, such as AlexNet, VGG, GoogLeNet and ResNet. Before the Google Brain team proposed the EfficientNet network architecture in 2019 [48], previous architectures achieved better performance by expanding one dimension of the CNN; unlike previous models, the EfficientNet network determines the optimal values of the three parameters by using neural architecture search techniques to simultaneously search the depth, width, and size of the model’s input image resolution. Compared with other networks, the EfficientNet model ensures higher accuracy while significantly reducing the parameter size of the model. Proposed by Google in 2021, EfficientNetV2 is an update of the EfficientNet architecture that offers better efficiency and smaller model parameters [49]. EfficientNet networks have been used to recognize and classify images in other fields and have achieved excellent performance [50,51,52,53,54]. Based on the high accuracy and small parameters of EfficientNet, especially the parameter size has unparalleled advantages compared to other models (hardware resources will be one of the main considerations for portable analytical instruments), we chose EfficientV2 as the experimental model. This study used the model to explore whether deep learning can detect specific substances within mixtures via the FAIMS system.

## 3. Results and Discussion

### 3.1. FAIMS Spectra

**Figure 3:**
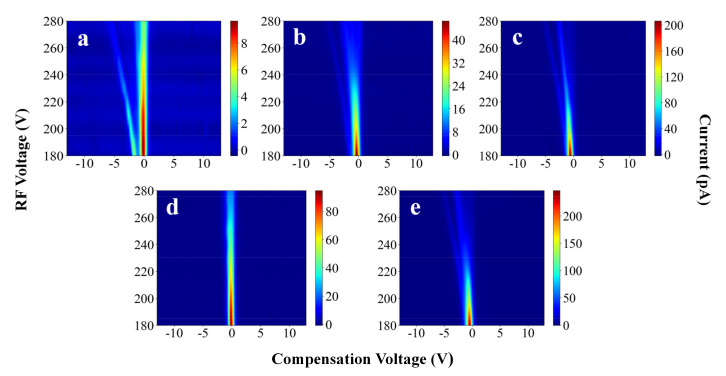
FAIMS spectra of pure compounds: (**a**) ethanol, (**b**) ethyl acetate, (**c**) acetone, (**d**) 4-methyl-2-pentanone, and (**e**) 2-butanone.

**Figure 4:**
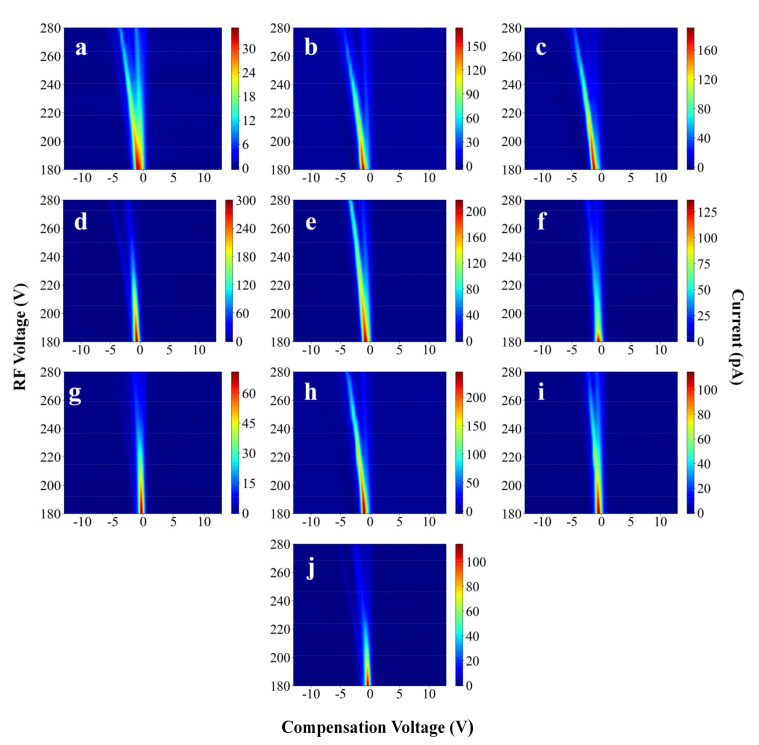
FAIMS spectra of several mixtures: (**a**) ethanol + ethyl acetate, (**b**) ethanol + acetone, (**c**) ethanol + acetone + ethyl acetate, (**d**) acetone + ethyl acetate, (**e**) ethanol + 4-methyl-2-pentanone, (**f**) ethanol + 4-methyl-2-pentanone + ethyl acetate, (**g**) 4-methyl-2-pentanone + ethyl acetate, (**h**) ethanol + 2-butanone, (**i**) ethanol + 2-butanone + ethyl acetate, and (**j**) 2-butanone + ethyl acetate.

The FAIMS spectra provide three-dimensional CV, RF voltage, and current data. Five chemicals commonly found in the detection field were selected for the experiment: ethanol, ethyl acetate, acetone, 2-butanone, and 4-methyl-2 pentanone. Their corresponding FAIMS spectra are shown in Figure 3. In fact, the recognition of pure compounds can be easily achieved, whether from the shape of the spectra or the design of the algorithm. The pure compounds were mixed and placed into FAIMS. The resulting spectra are shown in Figure 4, where the experiments considered are ethanol + ethyl acetate, ethanol + acetone, ethanol + acetone + ethyl acetate, acetone + ethyl acetate, ethanol + 4-methyl-2-pentanone, ethanol + 4-methyl-2-pentanone + ethyl acetate, and 4-methyl-2-2-butanone + ethyl acetate. The goal of this experiment was to explore whether the deep-learning model could specifically identify certain substances within a mixture. All the experimental conditions were the same as in Section 2.3 and Section 2.4.

Each sample’s color column range is different in Figure 3 and Figure 4 which is to better illustrate the differences among the samples. In the experiments, the color columns of the different samples were in a fixed range (0 pA–350 pA), which was favorable for the experimental results. The dataset is characterized in Table 1.

### 3.2. CNNs and FAIMS Spectra

FAIMS spectra can be viewed as images. Different substances produce different FAIMS spectra. Unlike traditional analytical discrimination methods, convolutional neural network methods preserve local two-dimensional features as well as sequential information. Since FAIMS spectral data are sequential in nature (any pixel in each row of the FAIMS spectra is related to the pixels before and after it) the use of CNNs for FAIMS spectral analysis is further justified. However, it should be noted that the dataset required to train a deep-learning model is huge. While it was not possible to provide such a huge dataset in this experiment, transfer learning provided a convenient path. Specifically, a model that was previously trained on a huge dataset was then trained using a smaller dataset. Within CNNs, different layers learn different image features. The shallower the layer, the more generic the features learned. The deeper the layer, the more relevant the features learned are to a specific task. Therefore, generic features can be pre-trained using a large dataset and the resulting model later trained for specific tasks [55]. Fine-tuning of pre-trained models is one way to implement migration learning [56]. In this study, the impacts of training a completely new model and using a pre-trained model are explored. The results show that a fine-tuned, pre-trained model provides better performance than a completely new model.

### 3.3. Experimental Results

Pure ethanol and mixtures that contain ethanol are classified as class 1 (serial numbers 1, 6, 7, 8, 11, 13, and 14 in Table 1) samples. Other pure compounds and mixtures that do not contain ethanol are classified as class 2 (serial numbers 2, 3, 4, 5, 9, 10, 12, and 15 in Table 1) samples. The same experiment was performed again by classifying the mixtures as above but first with ethyl acetate and then acetone taking the place of the ethanol. The dataset obtained according to this method is shown in Table 2. The EfficientNetV2 model was built using Python and the homemade dataset was used to train the model. Before feeding the data into the model, the dataset was divided into training, validation, and blind test sets. The training set was provided to the model in order to train the model parameters. The validation set was used to test the performance of the model in order to update the parameters. Finally, after a model that performed well on the validation set was obtained, a blind test set was used to simulate the real situation. The final results obtained using the blind test set were considered representative of the real situation [57]. The blind test set used in the experiment has 30 spectra, and its distribution is shown in the column “Blind test set number” in Table 1.

In this experiment, 80% of the data were used to train the model, 20% of the data were used to validate the model, and finally an additional blind test set of 30 images was used to test model performance. When the model produces an output of 0, the sample contains the substance of interest. In contrast, the sample does not contain the substance when the output is 1. The hyperparameters that affect the model include the learning rate (lr) and number of epochs. A smaller learning rate might improve model accuracy but can cause overfitting. In overfitting, the model performs well on the training set but poorly in the validation set [58]. Use of too many epochs increases the time required to train the model and use of too few epochs can lead to non-convergence of the model. It should be noted that different datasets have different optimal learning rates and the number of epochs (lr = 0.1 and epochs = 200 in this experimental dataset), must be determined on a task-specific basis. Due to the small dataset, standard five-fold cross-validation was used to verify model robustness. Briefly, five-fold cross-validation randomly divides all model datasets into five equal parts, where four portions are used to train the model and one portion is used to validate model performance [59]. Each fold will generate an accuracy rate, and finally, the average of the five results will be the most final evaluation metric of the model.

**Figure 5:**
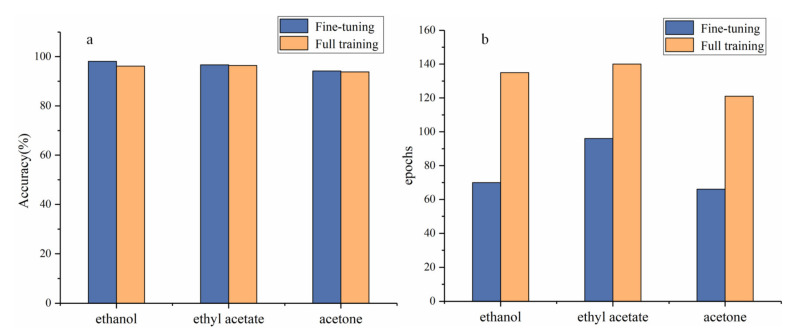
Results produced by fine-tuned and fully trained models. (**a**) Performance on the model validation set and (**b**) the number of epochs required for model convergence.

It is stated above that the use of pre-trained models is feasible for small-sample tasks. The impacts of full training and use of pre-trained models that are fine-tuned on the experimental task are explored in Figure 5. The pre-trained model with fine-tuning is more accurate than the model with full training. This shows the potential for the application of transfer learning to FAIMS spectral analysis and indicates a new path for future FAIMS spectral analysis tasks. The remainder of this paper references the pre-trained, fine-tuned model.

As shown in Figure 5a, the deep-learning model can achieve excellent performance on the dataset, regardless of whether we seek to distinguish ethanol, ethyl acetate, or acetone from the mixtures. Average accuracies of 98.1%, 96.7%, and 94.2% are obtained for ethanol, ethyl acetate, and acetone in the five-fold cross-validation study. This indicates that EfficientNetV2 is a robust algorithm that can detect specific substances within complex mixtures.

In order to simulate the performance of the model in real situations, the study used a blind test set to test the model. The blind test contained 30 FAIMS spectra, which were divided via the method used to construct the model. The results are shown in Table 3. Ethanol has the highest accuracy rate of 100%, ethyl acetate has almost the same accuracy rate as in the model validation set, and acetone exhibits a decrease of about 8%. This may be related to the small amounts of acetone and its mixtures in the samples. All samples exhibit an accuracy rate that exceeds 85%. A larger dataset may provide better results, but the data are sufficient to show that applying deep learning to a homemade FAIMS spectra dataset can enable the detection of specific substances in mixtures.

In addition, traditional analysis methods were used to test the data. A normalization operation was first performed on all images, which sped up the convergence of the model. PCA was used to downscale the images and select the top 50 principal components as features, and then an SVM model was used for classification. The results produced using the entire dataset are shown in Table 4.

Upon comparing Table 3 and Table 4, it can be seen that deep learning provides higher accuracy than traditional PCA + SVM analysis methods. This may be because the mechanisms that govern FAIMS are complex. Simple linear methods may not be sufficient to explain FAIMS spectra well. In Figure 3 and Figure 4, the FAIMS spectra of the different substances cannot be represented linearly. Neural networks offer better performance on nonlinear tasks. This is because neural networks can approximate arbitrary functions. Thus, neural networks are more suitable for FAIMS spectral analysis than linear methods. More importantly, deep learning does not require data preprocessing or manual extraction of subjectively important features. The model automatically extracts features, thus simplifying the use of FAIMS in practical applications.

In short, the experiments show that the deep-learning model can detect specific compounds within mixtures. In particular, the pre-trained model shows its potential for application to FAIMS spectral analysis. The pre-trained deep-learning model is more efficient and provides more accurate results than traditional FAIMS spectral analysis methods.

### 3.4. Application

One potential application of this method is for the detection of complex biological samples. Studies have already shown that diabetic patients have different levels of acetone in exhaled gas than healthy individuals, which can be used as a biomarker for diabetes [60,61]; dimethyl disulphide in the feces of cholera patients can be used as a biomarker [62,63], and trimethylaminuria patients have much higher levels of trimethylamine in their urine and sweat than healthy individuals [64,65]. Samples such as exhaled gas, urine, and feces contain a large number of different volatiles from which identifying specific disease markers would be a challenge, which may be possible using deep learning. There is also the promise of enabling prediction of disease marker concentrations, which can vary at different stages of disease, and we expect FAIMS to enable early prediction of disease using deep learning, which would be exciting.

## 4. Conclusions

This study used a deep-learning model to detect specific substances in complex mixtures via a homemade FAIMS system. This is the first application of deep learning to the analysis of FAIMS mixtures for component identification. It demonstrated the transfer learning techniques has potential for FAIMS spectra analysis. The fine-tuned EfficientNetV2 deep-learning model was used to test for the presence of ethanol, ethyl acetate, and acetone in complex mixtures using a self-constructed dataset. The results showed that the deep-learning model could identify specific substances in complex mixtures. In addition to providing better results than traditional methods, the deep-learning model did not require data pre-processing or manual feature extraction. This further improves the efficiency of FAIMS and, to a certain extent, removes human judgment from the process. This makes the results more objective.

In conclusion, this study used a pre-trained deep-learning model to identify specific substances within complex mixtures. Model performance is expected to improve as the dataset is expanded. This result is exciting with regard to future research. We can use deep learning to experiment on more mixtures and attempt to analyze complex biological samples. This will promote the development of the field of FAIMS spectral analysis. However, the model size remains an issue to be addressed. It might be solved by deploying the model in the cloud. Of course, this study is only a small step forward. In addition to substance characterization, future work may include the quantification of specific substances in mixtures, which can be achieved by collecting the FAIMS spectra of mixtures that contain different amounts of substances. Future work may also include further cloud deployment of the model and development of faster, more portable FAIMS systems.

## Figures and Tables

**Table 1:** Characterization of the experimental dataset.

Serial Number	Sample	Model Dataset Number	Blind Test Set Number
1	ethanol	18	2
2	ethyl acetate	13	2
3	acetone	18	2
4	4-methyl-2-pentanone	18	2
5	2-butanone	19	2
6	ethanol + ethyl acetate	18	2
7	ethanol + acetone	18	2
8	ethanol + acetone + ethyl acetate	18	2
9	acetone + ethyl acetate	17	2
10	ethanol + 4-methyl-2-pentanone	18	2
11	ethanol + 4-methyl-2-pentanone + ethyl acetate	18	2
12	4-methyl-2-pentanone + ethyl acetate	17	2
13	ethanol + 2-butanone	19	2
14	ethanol + 2-butanone + ethyl acetate	18	2
15	2-butanone + ethyl acetate	18	2
Total		265	30

**Table 2:** Data distribution.

Sample	Number of Class 1 Samples *	Number of Class 2 Samples	Total
ethanol	145	120	265
ethyl acetate	137	126	265
acetone	71	194	265

* Class 1 indicates pure compounds of interest to the model as well as mixtures containing the substance of interest, whereas class 2 indicates other pure compounds as well as mixtures that do not contain the substance.

**Table 3:** The performance of the blind test set.

Blind Test Set		Number of Real Labels	Number of Predicted Labels	Accuracy
1	other	14	14	100%
	ethanol	16	16	
2	other	14	13	96.7%
	ethyl acetate	16	17	
3	other	22	18	86.7%
	acetone	8	12	

**Table 4:** Performance of PCA and the SVM.

Sample	Training Set	Validation Set	Blind Dataset
ethanol	74.5%	79.2%	70.0%
ethyl acetate	81.5%	75.0%	66.7%
acetone	85.4%	78.8%	80.0%

## Data Availability

Not applicable.
